# A Multi-Ingredient Formula Ameliorates Exercise-Induced Fatigue by Changing Metabolic Pathways and Increasing Antioxidant Capacity in Mice

**DOI:** 10.3390/foods10123120

**Published:** 2021-12-16

**Authors:** Hui Chen, Xuan Ma, Lixing Cao, Shuang Zhao, Chong Zhao, Shutao Yin, Hongbo Hu

**Affiliations:** Department of Food Nutrition and Safety, College of Food Science and Nutritional Engineering, China Agricultural University, Beijing 100083, China; B20183060527@cau.edu.cn (H.C.); BS20193060576@cau.edu.cn (X.M.); bs20203060530@cau.edu.cn (L.C.); zaosuangh@cau.edu.cn (S.Z.); zhaoch0206@cau.edu.cn (C.Z.); yinshutao@cau.edu.cn (S.Y.)

**Keywords:** exercise-induced fatigue, formula, fat/glucose metabolisms, oxidative stress

## Abstract

Multiple mechanisms are involved in exercise-induced fatigue, including energy depletion, metabolite accumulation, and oxidative stress, etc. The mechanistic findings provide a rationale for a multi-targeted approach to exercise-induced fatigue management. This study created a multi-ingredient formula mixed with valine, isoleucine, leucine, β-alanine, creatine, l-carnitine, quercetin, and betaine, based on the functional characteristics of these agents, and evaluated the preventive effect of this mechanism-based formula on exercise-induced fatigue. Results showed that the 7-d formula supplement significantly increased the running duration time of mice by 14% and the distance by 20% in an exhaustive treadmill test, indicating that the formula could delay fatigue appearance and improve exercise performance. Mechanistically, the formula enhanced fatty acid oxidation and spared liver glycogen by regulating the fat/glucose metabolism-related signaling pathways, including phospho-adenosine monophosphate-activated protein kinase α (p-AMPKα), phospho-acetyl CoA carboxylase (p-ACC), carnitine palmitoyl-transferase 1B (CPT1B), fatty acid translocase (CD36), and glucose transporter type 4 (GLUT4), and increased antioxidant capacity. The findings suggested that the formula tested in this study effectively ameliorated exercise-induced fatigue by targeting multi-signaling pathways, showing promise as a regimen to fight exercise-induced fatigue.

## 1. Introduction

Exercise-induced fatigue is commonly defined as the inability of muscles to generate force due to exercise. The mechanisms involved in exercise-induced fatigue is not fully understood, but it is generally accepted that the leading causes of exercise-induced fatigue include energy depletion, metabolite accumulation, oxidative stress [1,2,3], increased muscle contraction-associated pro-inflammatory cytokines, deregulated neuro-immune-endocrine dysfunction, and altered hypothalamic-pituitary-adrenal axis activity [4]. Delayed exercise fatigue is often reflected by increased exercise endurance and better adaptation to energy metabolism and oxidative stress [5,6]. Excessive exercise fatigue affects work efficiency, leads to endocrine disorders, decreased immunity, and even organic diseases [7,8].

Nutritional intervention is suggested as a practical approach to exercise-induced fatigue management [9]. Dietary adenosine triphosphate (ATP), glucose, creatine, carnitine, glutamine, betaine, Nitric oxide (NO) can service as energy-providing or energy-boosting substances to combat against energy insufficiency-mediated fatigue. Vitamin C, vitamin E, Coenzyme Q (CoQ), and some phytochemicals such as quercetin, sulforaphane, curcumin, epigallocatechin gallate (EGCG), and resveratrol can function as antioxidants to mitigate oxidative stress-mediated muscle damage. Branched-chain amino acids (BCAAs) and caffeine can target neurotransmitter serotonin (5-HT) and dopamine (DA) to delay the onset of neurotransmitter-mediated central nervous system (CNS) fatigue. 

As mentioned above, energy depletion, oxidative stress, inflammation, and neurotransmitter-mediated action are the key events involved in exercise fatigue, and single-agent can only target partial events or signaling pathways involved in the exercise-induced exercise. It is reasonable to hypothesize that simultaneously targeting these multiple mechanisms by a combination of these agents might achieve a better efficacy against exercise-induced fatigue. This study created a multi-ingredient formula mixed with valine, isoleucine, leucine, β-alanine, creatine, l-carnitine, quercetin, and betaine, based on the functional characteristics of these agents (Table 1), and evaluated the preventive effect of this mechanism-based formula on exercise-induced fatigue using exhaustive treadmill test. We speculated that this mechanism-based formula would likely produce a strong anti-fatigue effect through concurrently targeting the key events involved in exercise-induced fatigue such as energy metabolism, oxidative stress, and inflammation. 

## 2. Materials and Methods

### 2.1. Chemicals and Reagents

The valine, isoleucine, leucine, β-alanine, creatine, l-carnitine, and betaine were purchased from Beijing Solarbio Science and Technology Co. Ltd. (Beijing, China), while the quercetin was obtained from Aladdin (Shanghai, China). The primary antibodies: rabbit anti-phospho-adenosine monophosphate-activated protein kinase α (p-AMPKα) (#2535S), rabbit anti-phospho-acetyl CoA carboxylase (p-ACC) (#11818S), mouse anti-glucose transporter type 4 (GLUT4) (#2213S), and rabbit anti-catalase (CAT) (#14097S) were purchased from Cell Signaling Technology (Beverly, MA, USA), while the rabbit anti-carnitine palmitoyl-transferase 1B (CPT1B) (ab134988), rabbit anti-fatty acid translocase (CD36) (ab252922), and mouse anti-glutamate-cysteine ligase catalytic subunit (GCLC) (ab55435) were purchased from Abcam (Cambridge, UK). The rabbit anti-superoxide dismutase (SOD2) (BS6734) was acquired from Bioworld (Beijing, China), and the rabbit anti-glyceraldehyde-3-phosphate dehydrogenase (GAPDH) was purchased from ABclonal (Boston, MA, USA). The GLUT4 (GB11244) used during immunofluorescence was provided by Servicebio (Wuhan, China). The secondary antibodies, namely horseradish peroxidase-linked goat anti-rabbit IgG (Cat#485) and horseradish peroxidase-linked goat anti-mouse IgG (Cat#330), were obtained from MBL (Woburn, MA, USA).

### 2.2. Animals and Treatments

The animal care and procedures were approved by the China Agricultural University Institutional Animal Care and Use Committee (the ethical approval code is RD-20214051-2). Male C57BL/6J mice (6 to 8 weeks old, 20–22 g) were purchased from Charles River Laboratories (Beijing, China) and housed in a temperature- (18–22 °C) and humidity-controlled (55–60%) environment, at a 12 h light/12 h dark cycle for a week to acclimatize. All animals were allowed ad libitum access to standard chow (Beijing Keaoxieli Co. Ltd., Beijing, China) and water. 

**Figure 1:**
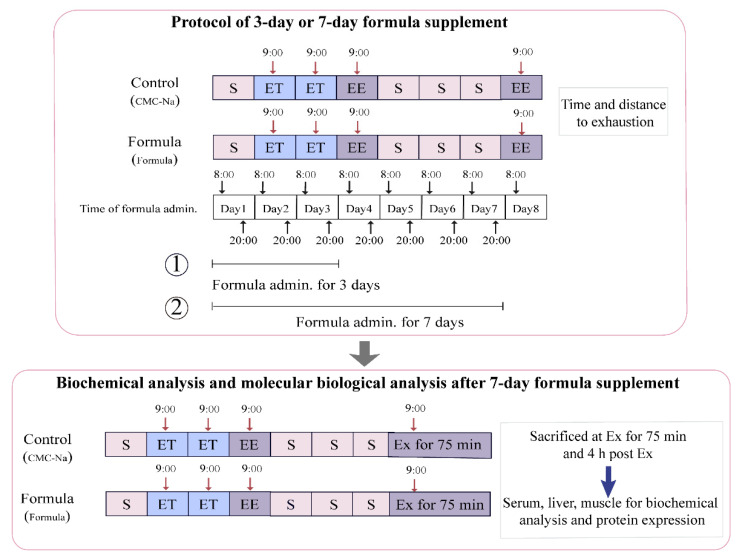
A schematic representation of the experimental design. Control: This refers to the control group, consisting of mice who were administered a 0.5% CMC-Na solution intragastrically and also received ET and EE. Formula: This refers to the formula group, consisting of mice who were administered an indicative dose of the formula intragastrically, as well as ET and EE. Ex: exercise. Ex for 75 min: This refers to exercise for 75 min according to the protocol of the exhaustive treadmill test. 4 h post-Ex: This refers to 4 h post-exercise for 75 min. S: sedentary; ET: exercise training; EE: exhaustive exercise. ①: The schedule of the 3-d formula supplementation, followed by EE. ②: The schedule of the 7-d formula supplementation, followed by EE.

The multi-ingredient formula containing valine, isoleucine, leucine, β-alanine, creatine, l-carnitine, quercetin, and betaine was pre-mixed and then suspended in the 0.5% CMC-Na solution (CMC-Na: distilled water = 0.5:100, *w*/*v*). The total daily dose of each component was 125 mg/kg, 125 mg/kg, 250 mg/kg, 182 mg/kg, 250 mg/kg, 200 mg/kg, 25 mg/kg, and 284 mg/kg body weight, corresponding to valine, isoleucine, leucine, β-alanine, creatine, l-carnitine, quercetin, and betaine, respectively, which was based on the available literature [4,10,11,12,13,14,15]. The formula was administrated twice a day at half the total daily dose via oral gavage at 8:00 am and 8:00 pm for 3 d and 7 d, respectively, after which an exhaustive treadmill test was performed to assess the acute effect of the formula on exercise performance, the volume of oral-gavage was 15 mL/kg body weight. The two timelines of 3-d or 7-d supplement were commonly applied in the evaluation of acute anti-fatigue function [6,14,17]. The entire study design is shown in Figure 1, and is divided into two parts:

The first involved the exhaustive treadmill test. The formula was administrated intragastrically 1 h before the test. The mice treated with 0.5% CMC-Na solution instead of formula were regarded as the control group. For the 3-d supplement, 12 mice from either the control or the formula group were used for the exhaustive treadmill test at 4 d. For the 7-d supplement, nine mice from the control group and 12 mice from the formula group were used for the exhaustive treadmill test at 8 d. 

The second category involved the biochemical and molecular biological analyses. The exhaustive treadmill test results indicated that the 7-d formula supplement significantly alleviated exercise-induced fatigue and was consequently selected to further explore the anti-fatigue mechanisms. Another batch of mice, consisting of 12 from the control group and 12 from the formula group, were used for this experiment. After an exercise period of 75 min (Ex for 75 min), six mice from each group were sacrificed immediately, followed by the rest at 4 h post-exercise (4 h post-Ex). Then the blood, muscles, and livers were collected for subsequent analysis.

### 2.3. Exhaustive Treadmill Test

**Figure 2:**
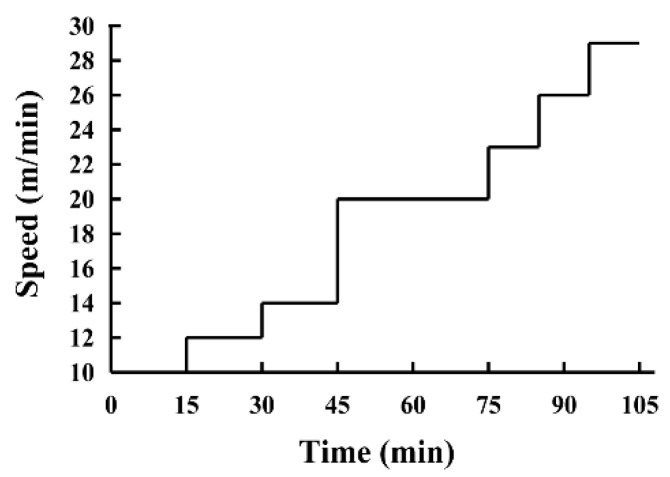
The protocol of the exhaustive treadmill test.

The exhaustive treadmill test was performed using an FT-200 animal treadmill (Chengdu Techman Software Co. Ltd., Chengdu, China), testing endurance with high-intensity ramping exercise, using a method described by Seiler et al. [15] with minor modifications. The exercise program was implemented as follows: 3 d prior to the exhaustive treadmill test, the mice were acclimated via exposure to a 3 min run for 1 min at 10 m/min, 12 m/min, and 14 m/min, respectively. During the test, the incline of the treadmill was fixed at 10°. The mice began running at 10 m/min, after which the speed increased with 2 m/min every 15 min for 45 min before transitioning to 20 m/min. This speed was maintained for 30 min, after which it was increased to 23 m/min for 10 min, followed by an increase of 3 m/min every 10 min until exhaustion (Figure 2). Exhaustion was defined as remaining on the shock grid for more than 10 s accompanied by nudging. The time and distance required to reach exhaustion were recorded to reflect the exercise capacity of each mouse.

### 2.4. Sample Collection

All the mice were sacrificed via cervical dislocation under diethylether anesthesia at Ex for 75 min and 4 h post-Ex. Blood was collected from the eyes and centrifuged at 3500 rpm for 10 min at 4 °C to isolate the serum. The livers, as well as the gastrocnemius and soleus muscles, were dissected as soon as possible and frozen in liquid nitrogen. All samples were stored at −80 °C pending analysis. 

### 2.5. Biochemical Serum and Tissue Analysis

The lactate dehydrogenase (LDH), creatine phosphokinase (CK), malondialdehyde (MDA) levels in the serum samples were analyzed using commercial kits from the Nanjing Jiancheng Bioengineering Institute (Nanjing, China) according to the protocols of the manufacturer.

The free fatty acid (FFA) content was assessed using commercial kits obtained from Beijing Solarbio Science and Technology Co. Ltd. (Beijing, China). The glycogen content in the livers and muscles and the antioxidant activity indexes of the gastrocnemius muscles, including MDA, CAT, and glutathione (GSH), were measured using a commercial kit from Nanjing Jiancheng Bioengineering Institute, following the instructions of the manufacturer.

### 2.6. Western Blot Analysis

Western blotting was performed according to a method delineated by Yan et al. [21] with minor modifications. The gastrocnemius muscles were lysed with radio-immunoprecipitation assay (RIPA) buffer. Forty micrograms of denatured proteins were subjected to sodiumdodecylsulfate (SDS)-polyacrylamide gel electrophoresis (PAGE) and transferred onto a polyvinylidene fluoride (PVDF) membrane, after which it was blocked with 5% non-fat dry milk-TBST buffer (TBS/Tween-20 1000:1, *v*/*v*) at room temperature. The membranes were incubated with specific antibodies to perform immunoblot analyses. The primary antibodies included p-AMPKα (1:1000), p-ACC (1:1000), CPTIB (1:1000), CD36 (1:1000), GLUT4 (1:1000), SOD2 (1:1000), CAT (1:1000), GCLC (1:1000), and GAPDH (1:5000), and were all diluted with TBST with 1% bovine serum albumin (BSA) to the appropriate concentration. The secondary antibodies included horseradish peroxidase-linked goat anti-rabbit IgG and horseradish peroxidase-linked goat anti-mouse IgG. The immunoreactive blots were detected via enhanced chemiluminescence, while X-ray films were used to record the signal. The films were scanned into a computer to obtain the images.

### 2.7. Immunofluorescence of the Muscle Paraffin Sections for GLUT4 Analysis 

GLUT4 staining was performed using 5 μm paraffin sections obtained from the gastrocnemius muscles of the Ex for 75 min mice in the control and formula groups. The tissue sections were incubated overnight at 4 °C with the diluted rabbit anti-GLUT4 antibody (1:200; Servicebio). The cell nucleus was marked with 4′,6-diamidino-2-phenylindole (DAPI), after which the CY3-conjugated goat anti-rabbit IgG secondary antibody (1:300; Servicebio) was used to detect the GLUT4 primary antibody. Images were captured using an upright fluorescence microscope (Nikon Eclipse C1; Nikon DS-U3 Microsystems, Nikon, Japan) at 400× magnification. The CY3 fluorophores were excited at 510–560 nm excitation and 590 nm emission, while the DAPI was excited at 330–380 nm and 420 nm.

### 2.8. Statistical Analysis

The data were expressed as mean ± standard deviation (mean ± SD) of the number of animals used in each experiment. The statistical analysis was performed using Duncan’s new multiple range test and DPS 7.5 software. Significance was indicated by *p* < 0.05, while Graph Pad Prism 7.00 software was used for graph plotting.

## 3. Results

### 3.1. The Formula Supplement Prolongs the Time and Running Distance Required to Reach Exhaustion during the Exhaustive Treadmill Test

**Figure 3:**
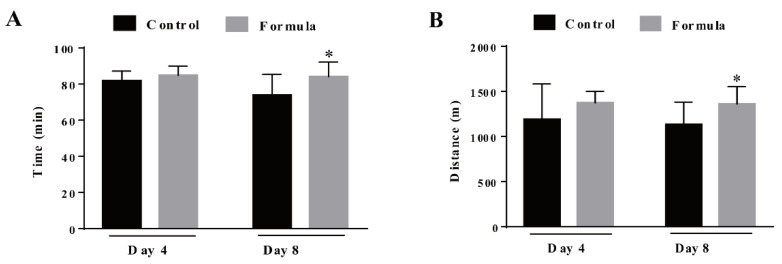
The time (**A**) and distance (**B**) required to reach exhaustion, measured during the exhaustive treadmill test in the control and formula groups. At 4 d, N = 12 mice in each group, at 8 d, N = 9 mice in the control group, and N = 12 mice in the formula group. Black column: control group and gray column: formula group. Data are expressed as mean ± SD, * *p* < 0.05, compared to the control group.

The mice received the formula supplement for either 3 d or 7 d, and their exercise performance was assessed by measuring the time and distance required to reach exhaustion. As shown in Figure 3, formula supplementation for 3 d slightly prolonged the time (prolonged by 4%) and running distance (increased by 6%) necessary to reach exhaustion, displaying no significant differences compared with the control group (*p* > 0.05). However, after the 7-d supplementation, the exercise endurance of mice in the formula group was significantly enhanced, increasing the exercise time by 14% (*p* = 0.0258) and the distance by 20% (*p* = 0.0302) (Figure 3A,B). These results indicate that the formula effectively mitigated exercise-induced fatigue in the current experimental conditions.

### 3.2. The Formula Supplement Induces Glucose and Lipid Metabolism Changes in Response to Acute Exercise

**Figure 4:**
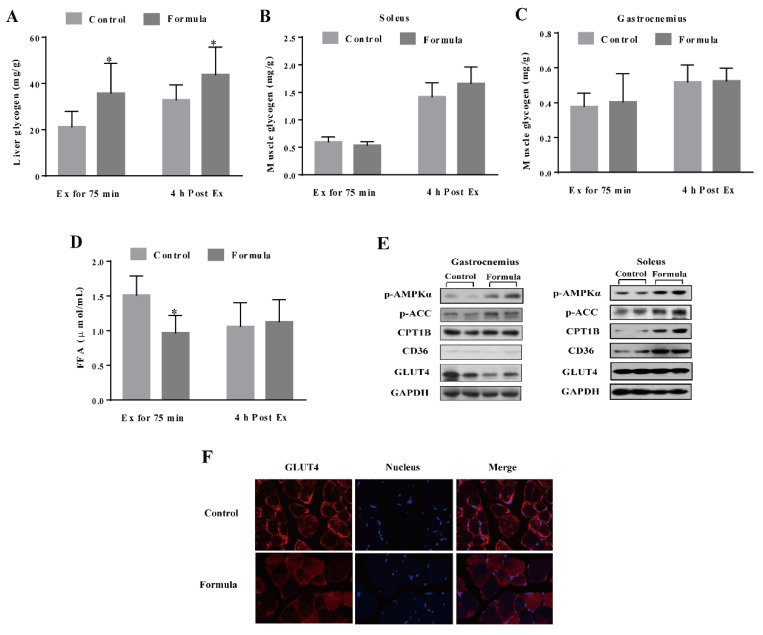
The level of energy substrates glycogen in the liver (**A**), soleus (**B**), and gastrocnemius (**C**) muscles and serum FFA (**D**) in each group after Ex for 75 min and 4 h post Ex for comparison among groups at the same time course. The protein expression in the glucose and fatty acid metabolism-related pathways in the gastrocnemius and soleus muscles in each group after Ex for 75 min (**E**). Immunofluorescence staining of the GLUT4 in the gastrocnemius muscles in each group after Ex for 75 min (**F**). Images were captured at the 400× magnification. Scale bar = 50 μm. The GLUT4 is shown in red, while the nucleus is depicted in blue. Ex: exercise. Ex for 75 min: Exercise for 75 min according to the protocol of the exhaustive treadmill test. 4 h post-Ex: 4 h after completion of the exercise for 75 min. FFA: free fatty acids; p-AMPKα: phospho-adenosine monophosphate-activated protein kinase α; p-ACC: phospho-acetyl CoA carboxylase; CPT1B: carnitine palmitoyl-transferase 1B; CD36: fatty acid translocase; GLUT4: glucose transporter type 4; GAPDH: glyceraldehyde-3-phosphate dehydrogenase. The light gray column denotes the control group, while the deep gray column represents the formula group. The data are expressed as mean ± SD, N = 6, * *p* < 0.05, compared to the control group at the same time.

As shown in Figure 4A, the liver glycogen level was significantly increased in the formula group compared with the control group at either Ex for 75 min or 4 h post Ex, while no significant differences were evident between the glycogen content in the soleus and gastrocnemius muscles of these two groups (Figure 4B,C). After Ex for 75 min, the formula group mice showed less serum FFA than those in the control group. Although a significant difference was evident, no such changes were found 4 h post-Ex (Figure 4D). In addition, the changes in the glucose/fat metabolism-related signaling pathways were shown in Figure 4E. The formula supplement obviously increased the AMPK and ACC phosphorylation in both the gastrocnemius and soleus muscles while upregulating the CPT1B and CD36 expression without affecting GLUT4 expression. However, immunofluorescence analysis of the GLUT4 distribution in the gastrocnemius muscle showed a clear visual redistribution to the cell membrane in the control group rather than the formula group (Figure 4F). Therefore, these findings suggested that the formula supplement caused metabolic changes from glucose utilization to fatty acid oxidation.

### 3.3. The Formula Supplement Ameliorates Exercise-Induced Oxidative Stress 

**Figure 5:**
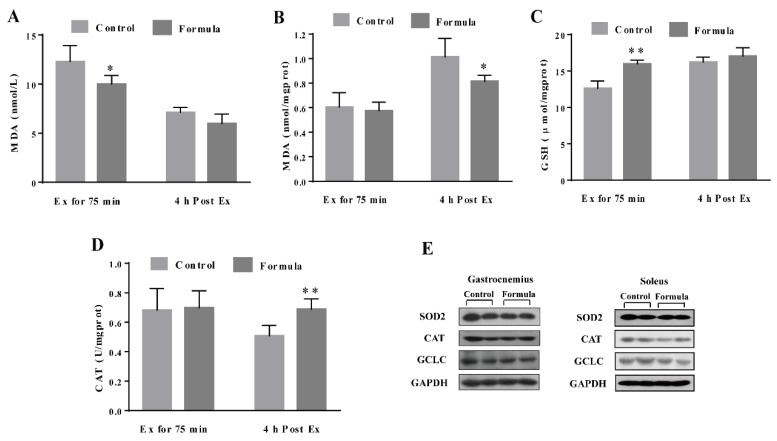
The levels of oxidative stress markers including serum MDA (**A**), and the MDA (**B**), GSH (**C**), and CAT (**D**) in the gastrocnemius muscles of each group after Ex for 75 min and 4 h post Ex for comparison among groups at the same time course. The protein expression in the antioxidant-related pathways in the gastrocnemius and soleus muscles of each group after Ex for 75 min (**E**). Ex: exercise. Ex for 75 min: Exercise for 75 min according to the protocol of the exhaustive treadmill test. 4 h post-Ex: 4 h after completion of the exercise for 75 min. MDA: malondialdehyde; GSH: glutathione; CAT: catalase; GCLC: glutamate-cysteine ligase catalytic subunit; SOD2: Superoxide dismutase 2; GAPDH: glyceraldehyde-3-phosphate dehydrogenase. The light gray column refers to the control group, while the deep gray column denotes the formula group. The data are expressed as mean ± SD, N = 6, * *p* < 0.05, and ** *p* < 0.01, compared to the control group at the same time.

**Figure 6:**
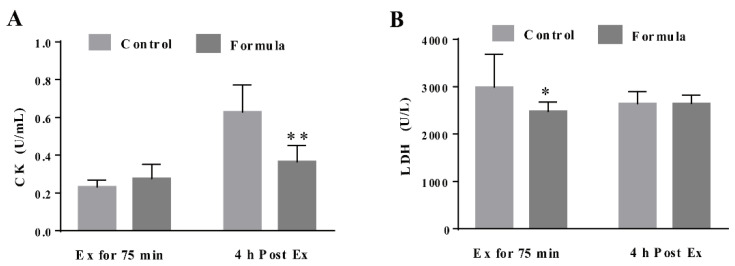
The injury-related biochemical parameters, CK (**A**) and LDH (**B**), in the serum of each group after Ex for 75 min and 4 h post Ex for comparison among groups at the same time course. Ex: exercise. Ex for 75 min: Exercise for 75 min according to the protocol of the exhaustive treadmill test. 4 h post-Ex: 4 h after completion of the exercise for 75 min. The data are expressed as mean ± SD, N = 6, * *p* < 0.05, and ** *p* < 0.01, compared to the control group at the same time.

To determine the contribution of oxidative stress inhibition to the formula-mediated anti-fatigue effect, the changes in several key oxidative stress biomarkers were measured, including the serum MDA, as well as the MDA, GSH, and CAT levels in the gastrocnemius muscles in response to exercise. As shown in Figure 5A,B, the serum MDA content in the formula group was significantly lower at Ex for 75 min and 4 h post Ex than in the control group. The GSH levels in the gastrocnemius muscles of the formula group were significantly higher than in the control group at Ex for 75 min (Figure 5C), while the formula supplement significantly enhanced CAT activity at 4 h post-Ex (Figure 5D). Therefore, formula supplementation decreased the oxidative stress caused by EE while displaying no significant effect on either CAT protein expression of SOD2 and GCLC levels (Figure 5E). Furthermore, oxidative stress supposedly causes muscle damage. Next, the changes in the CK and LDH levels were analyzed, representing the two key markers of muscle damage. As shown in Figure 6A, the CK levels were significantly lower in the formula group than in the control group at 4 h post-Ex, while the LDH levels in the formula group were substantially lower in the formula group than in the control group at Ex for 75 min (Figure 6B). The data suggested that the formula supplement suppressed exercise-induced oxidative stress, which in turn protected against exercise-induced muscle damage.

## 4. Discussion

Exercise fatigue is a multiple mechanism-mediated phenomena. Simultaneously targeting these mechanisms is a reasonable approach for improving exercise performance. This study evaluated a formula consisting of valine, isoleucine, leucine, β-alanine, creatine, l-carnitine, and betaine, and assessed the preventive effect on exercise-induced fatigue. The results demonstrated that continuously exposing mice to the formula supplement for 7 d significantly prolonged the exercise time and distance required to reach exhaustion during the exhaustive treadmill test, through changing metabolic pathways and increasing antioxidant capacity in mice. The data indicated that this mechanism-based combination regimen is an effective approach for combatting exercise-induced fatigue.

Glucose and fat represent the primary substrates of energy metabolism during exercise. To determine the contribution of each, this study analyzed the changes in the liver/muscle glycogen and serum FFAs in response to formula supplementation. Liver glycogen represents a vital substrate store for energy metabolism. Muscle glycogen is rapidly broken down into glucose during exercise to meet the muscle contraction requirements. When the muscle glycogen is depleted, liver glycogenolysis increases to sustain the glucose supply via the liver glycogen-blood glucose-muscle glycogen axis [22,23]. Studies have shown that liver glycogen sparing is positively associated with exercise performance [24]. This study demonstrated that the formula increased liver glycogen while decreasing the serum fatty acid content (Figure 4), suggesting that the formula can spare liver glycogen via a shift from glucose to fat in the fuel section (substrate utilization). In support of this notion, the formula initiated AMPK activation, evidenced by increased phosphorylation and the inhibition of its downstream target, ACC, which plays a crucial role in mitochondrial substrate selection for fatty acid oxidation by regulating the pyruvate dehydrogenase (PDH) complex, especially for its catalytic α2-subunit [25]. In agreement with the elevated fatty acid oxidation, CD36, a membrane transporter for fatty acids [26], and CPT1B, a rate-limiting enzyme for fatty acid oxidation in muscle mitochondria [27] were up-regulated in conjunction with decreased GLUT4 translocation in the gastrocnemius muscles of the formula group, compared with the control group. The data obtained in this study suggested that a shift in energy substrate utilization contributed to the ability of the formula to improve exercise performance. 

It has been reported that l-carnitine, quercetin has a positive effect on promoting fatty acid oxidation. l-carnitine plays an essential role in the translocation of long-chain fatty acids into the mitochondrial matrix for subsequent β-oxidation [28]. A recent report shows that six weeks of dietary quercetin supplementation promotes glycogen storage and enhances the mRNA expression of regulators in muscle mitochondrial fatty acid β-oxidation, such as β-hydroxyacyl coenzymes A dehydrogenase (HADH), carnitine palmitoyltransferase 1 (CPT1), peroxisome proliferator-activated receptor-delta (PPARδ), uncoupling protein-3 (UCP3) [29]. Another report confirmed that quercetin enhances fatty acid β-oxidation in AML12 hepatocytes by inducing lipophagy [30]. Therefore, it is considered that the formula supplement-induced a shift in fuel section (substrate utilization) from glucose toward fat could be mediated by l-carnitine and quercetin. In addition, betaine is one of the sources for carnitine endogenous synthesis [31], so it is likely that betaine was transformed into l-carnitine in vivo and then promoted fatty acids oxidation. In addition, BCAAs might serve as sources of hepatic gluconeogenesis [32], and creatine is directly metabolized to produce energy during exercise, which in turn contributed to the upregulation of liver glycogen levels in formula supplements.

Muscular contraction and intense exercise increase reactive oxygen species (ROS) production and promote oxidative stress in skeletal muscle, which has been implicated in muscle damage [33]. Improving the antioxidant capacity is considered an effective preventative approach to fight against exercise-induced fatigue. Several oxidative stress-related markers were examined, and the results showed increased antioxidant capacity in the formula group, evidenced by a lower MDA level, as well as higher GSH levels and CAT activity in the muscles (Figure 5). In support of oxidative stress amelioration, the key muscle damage marker, CK, and the LDH level [34,35] were reduced in response to the formula treatment (Figure 6). However, no significant effect was evident on the antioxidant protein expression after formula supplementation. This further demonstrated that the formula directly reduced ROS generation or modified some antioxidant enzyme activities to inhibit exercise-induced oxidative stress. Quercetin has been reported to scavenge free radicals directly [36]. Furthermore, Chen et al. [29] found that quercetin treatment decreased the MDA content and enhanced CAT activities in serum and gastrocnemius muscle of mice who experienced non-loading swimming tests. β-alanine is a rate-limiting factor for intramuscular carnosine synthesis. Due to its low concentration in muscle, a supplement of exogenous β-alanine can increase carnosine content in muscle [11,37]. Carnosine is a well-known anti-oxidative dipeptide to alleviate oxidative stress by direct or indirectly scavenging free radicals [38]. l-carnitine not only plays a central role in fatty acid metabolism but also acts as an antioxidant. A growing number of studies have shown that l-carnitine can boost the activity of endogenous antioxidants, reduce lipid peroxide, and remove reactive oxygen species (ROS) in different oxidative stress models in vitro/vivo, including exhaustive exercise [39]. Collectively, quercetin, β-alanine, and l-carnitine may play a central role in reducing excessive oxidative stress-induced muscle damage and then improve exercise performance. 

## 5. Conclusions

This study investigates the effect of a formula supplement containing valine, isoleucine, leucine, β-alanine, creatine, l-carnitine, quercetin, and betaine on exercise-induced fatigue. The main findings are as follows: First, after 7 d of formula supplementation, the exercise endurance of the mice is significantly enhanced. Second, formula supplementation increases the proportion of fatty acids as a source of metabolic fuel supporting energy production and preserving liver glycogen during exercise. Third, formula supplementation protects against muscle damage from the oxidative stress generated by EE. In summary, the formula ameliorates exercise-induced fatigue and is mechanistically associated with the substrate shift from glucose to fatty acids and increasing antioxidant capacity. However, whether the formula ingredients have a synergistic or accumulation effect on relieving fatigue requires further investigation. The findings suggest that simultaneously targeting the diverse mechanisms involved in exercise-induced fatigue may be a practical and effective preventative approach. Furthermore, this mechanism-based formula shows promise to be developed as an effective remedy for exercise fatigue management.

## Figures and Tables

**Table 1:** Functional characteristics of the ingredients in the formula supplement.

Ingredients	Functional Characteristics	References
BCAAs (Valine, isoleucine and leucine)	Reduces the formation of 5-hydroxytryptamine, delays central fatigue, protects muscle damage, energy substrates for long-term endurance exercise inhibits muscle lactate production, reduces serum creatine level, promotes adipose decomposition (isoleucine), and stimulates muscle protein synthesis (leucine).	[4,10]
β-alanine	Involved in carnosine synthesis and enhances the total buffering ability of the skeletal muscles.	[11,12]
Creatine	Rapidly supplies energy in a short time, improves glucose uptake, increases muscle glycogen accumulation, acts as an H^+^ buffer, and promotes aerobic metabolism.	[13]
L-carnitine	Promotes fatty acids oxidation, enhances the antioxidant effect, improves glucose tolerance, reduces the blood lactate level, improves maximum oxygen consumption by the body, and alleviates muscle injury.	[14,15]
Quercetin	Enhances the antioxidant effect, promotes mitochondrial synthesis, reduces protein or amino acid consumption, increases fat mobilization, protects mitochondrial functionality, and improves energy metabolism.	[16,17,18]
Betaine	Increases the creatine synthesis, improves the nitric oxide level in the blood, increases blood flow, stimulates lipid decomposition, inhibits adipogenesis, stimulates the release of autocrine/endocrine IGF-1 and insulin receptor signaling pathways, stimulates growth hormone secretion, and increases protein synthesis.	[19,20]

## Data Availability

Data is contained within the article.

## Abbreviations

ATP, adenosine triphosphate; NO, Nitric oxide; CoQ, Coenzyme Q; EGCG, epigallocatechin gallate; BCAAs, Branched-chain amino acids; 5-HT, Serotonin; DA, dopamine; CNS, central nervous system; p-AMPKα, phospho-adenosine monophosphate-activated potein kinase α; p-ACC, phospho-acetyl CoA carboxylase; CPT1B, carnitine palmitoyltransferase 1b; CD36, fatty acid translocase; GLUT4, glucose transporter type 4; CAT, catalase; GCLC, glutamatecysteine ligase catalytic subunit; SOD2, Superoxide dis-mutase 2; GAPDH, glyceraldehyde-3-phosphate dehydrogenase; LDH, Lactate dehy-drogenase; CK, creatine phosphokinase; MDA, malondialdehyde; FFA, free fatty acids; CAT, catalase; GSH, glutathione; PDH, pyruvate dehydrogenase; HADH, β-hydroxyacyl coenzymes A dehydrogenase; CPT1, carnitine palmitoyltransferase 1; PPARδ, peroxisome proliferator-activated receptor-delta; UCP3, uncoupling protein-3; ROS, reactive oxygen species.
